# Supplementary material for: Modeling glioblastoma heterogeneity as a dynamic network of cell states
Source: Mol Syst Biol. 2021 Sep 16;17(9):e10105. doi: 10.15252/msb.202010105 (PMC8444284; doi:10.15252/msb.202010105)
Supplement: Supplementary file 6 — Source Data for Figure 5 [file MSB-17-e10105-s004.zip › Figure5A_sourcedata/GSEA_3017/hallmarks_stateF.GseaPreranked.1621934628066/HALLMARK_IL6_JAK_STAT3_SIGNALING.html]

Details for gene set HALLMARK\_IL6\_JAK\_STAT3\_SIGNALING[GSEA]

|  || Dataset | state33017 |
| Phenotype | NoPhenotypeAvailable |
| Upregulated in class | na\_pos |
| GeneSet | HALLMARK\_IL6\_JAK\_STAT3\_SIGNALING |
| Enrichment Score (ES) | 0.47606024 |
| Normalized Enrichment Score (NES) | 1.808744 |
| Nominal p-value | 0.005628518 |
| FDR q-value | 0.017724838 |
| FWER p-Value | 0.19 |
Table: GSEA Results Summary

  

Fig 1: Enrichment plot: HALLMARK\_IL6\_JAK\_STAT3\_SIGNALING      
 Profile of the Running ES Score & Positions of GeneSet Members on the Rank Ordered List

  

| PROBE | GENE SYMBOL | GENE\_TITLE | RANK IN GENE LIST | RANK METRIC SCORE | RUNNING ES | CORE ENRICHMENT || 1 | TNFRSF21 |  |  | 36 | 1.016 | 0.1012 | Yes |
| 2 | A2M |  |  | 42 | 0.974 | 0.2114 | Yes |
| 3 | HMOX1 |  |  | 142 | 0.684 | 0.2460 | Yes |
| 4 | CD44 |  |  | 191 | 0.600 | 0.2939 | Yes |
| 5 | PDGFC |  |  | 219 | 0.577 | 0.3484 | Yes |
| 6 | STAT2 |  |  | 267 | 0.540 | 0.3897 | Yes |
| 7 | IL6ST |  |  | 321 | 0.500 | 0.4238 | Yes |
| 8 | CSF1 |  |  | 333 | 0.495 | 0.4761 | Yes |
| 9 | TNFRSF1A |  |  | 686 | 0.374 | 0.3619 | No |
| 10 | FAS |  |  | 740 | 0.361 | 0.3799 | No |
| 11 | SOCS3 |  |  | 869 | 0.332 | 0.3610 | No |
| 12 | STAT3 |  |  | 880 | 0.330 | 0.3946 | No |
| 13 | IRF9 |  |  | 892 | 0.327 | 0.4274 | No |
| 14 | CD9 |  |  | 1070 | 0.297 | 0.3826 | No |
| 15 | OSMR |  |  | 1111 | 0.291 | 0.3983 | No |
| 16 | IFNGR1 |  |  | 1289 | 0.269 | 0.3503 | No |
| 17 | TNFRSF12A |  |  | 2116 | -0.694 | 0.0612 | No |
Table: GSEA details [plain text format]

  

Fig 2: HALLMARK\_IL6\_JAK\_STAT3\_SIGNALING: Random ES distribution      
 Gene set null distribution of ES for **HALLMARK\_IL6\_JAK\_STAT3\_SIGNALING**
